# Automatic Tracking Based on Weighted Fusion Back Propagation in UWB for IoT Devices †

**DOI:** 10.3390/s24041257

**Published:** 2024-02-16

**Authors:** Boliang Zhang, Lu Shen, Jiahua Yao, Tenglong Wang, Su-Kit Tang, Silvia Mirri

**Affiliations:** 1 Faculty of Applied Sciences, Macao Polytechnic University, Macao SAR, China; 2 College of Financial Technology, Shenzhen University, Shenzhen 518060, China; 3 Department of Computer Science and Engineering, University of Bologna, Mura Anteo Zamboni, 7, 40124 Bologna, Italy

**Keywords:** Internet of Things, UWB jitter value, BP neural network, follow algorithm, hybrid filtering, indoor localization

## Abstract

The global population is progressively entering an aging phase, with population aging likely to emerge as one of the most-significant social trends of the 21st Century, impacting nearly all societal domains. Addressing the challenge of assisting vulnerable groups such as the elderly and disabled in carrying or transporting objects has become a critical issue in this field. We developed a mobile Internet of Things (IoT) device leveraging Ultra-Wideband (UWB) technology in this context. This research directly benefits vulnerable groups, including the elderly, disabled individuals, pregnant women, and children. Additionally, it provides valuable references for decision-makers, engineers, and researchers to address real-world challenges. The focus of this research is on implementing UWB technology for precise mobile IoT device localization and following, while integrating an autonomous following system, a robotic arm system, an ultrasonic obstacle-avoidance system, and an automatic leveling control system into a comprehensive experimental platform. To counteract the potential UWB signal fluctuations and high noise interference in complex environments, we propose a hybrid filtering-weighted fusion back propagation (HFWF-BP) neural network localization algorithm. This algorithm combines the characteristics of Gaussian, median, and mean filtering, utilizing a weighted fusion back propagation (WF-BP) neural network, and, ultimately, employs the Chan algorithm to achieve optimal estimation values. Through deployment and experimentation on the device, the proposed algorithm’s data preprocessing effectively eliminates errors under multi-factor interference, significantly enhancing the precision and anti-interference capabilities of the localization and following processes.

## 1. Introduction

In recent years, with the rapid development of technology, the demand for precise positioning techniques has been increasingly growing [1]. Against this backdrop, autonomous positioning and following Internet of Things (IoT) devices have gradually come into the public eye. However, these devices still face numerous challenges in positioning accuracy and adaptability to complex environments, often leading to suboptimal following performance and functionality. Although the market is not short of products capable of autonomous positioning and following, they typically struggle to achieve flexible and precise positioning and following, especially in varied and complex environments. Concurrently, the global population is progressively aging, with the number and proportion of elderly individuals rising in almost every country. Existing autonomous positioning and following IoT devices often overlook the unique needs of vulnerable groups such as the disabled, elderly, and pregnant women, urgently necessitating solutions for stable tracking and assistance in handling objects in complex terrains like stairs, plaza steps, and campuses. Moreover, the inevitable Non-Line of Sight (NLOS) issues in real-world environments significantly impact positioning accuracy [2,3,4,5,6]. In this context, Ultra-Wideband (UWB) technology, known for its excellent penetration capabilities, low power consumption, and high positioning accuracy, has attracted widespread attention in high-precision positioning [7].

In the current market landscape, autonomous following robots predominantly utilize visual sensor technology to track moving targets. However, these vision-based following methods are susceptible to lighting conditions, prone to interference from external light sources, and often lose track of their target [8,9]. In environments such as indoors, train stations, and airports, multipath and NLOS interferences impede effective positioning and following. Jinhai Liu et al. [10] designed a cart that autonomously follows a target using UWBxuzo technology, yet UWB signal fluctuations significantly disrupt the positioning in complex environments. Qingkun Song et al. [11] attempted to mitigate indoor multipath and NLOS effects using Kalman filters for range data processing, but the results were suboptimal. To enhance positioning accuracy, Bi J et al. [12] proposed an indoor WiFi positioning algorithm based on Particle Swarm Optimization-Support Vector Regression. Their findings indicated a reduction in Root-Mean-Squared Error (RMSE) compared to methods based on Convolutional Neural Networks. Edgar S et al. [13] introduced an improved hybrid technique that combines received signal strength information from available WLAN access points with wireless sensor network technology for fingerprint-based indoor positioning, effectively reducing positioning errors. Peng S et al. [14] developed an algorithm that integrates Weighted K-nearest neighbors and Kalman Filtering, proposing enhancements like weighted K-nearest neighbor Particle filtering and weighted K-nearest neighbor extended Kalman filtering to lower positioning errors effectively. Yue D et al. [15] proposed a navigation localization model based on an improved-bee-algorithm-optimized BP network. They utilized the IABC algorithm and a Kalman filter to establish a navigation localization model for agricultural machinery BDS/INS, achieving reliable navigation and precise positioning in a short time. Guoping D et al. [16] employed the GWO algorithm to optimize a BP neural network, constructing a CFRP transmission shaft damage identification and localization system based on FBG sensor networks and GWO-BP neural networks. This system enables real-time acquisition of CFRP transmission rod strain field information, accurately identifying damage and its location. The Chan algorithm, known for its low computational demand, is significantly affected by NLOS errors. The presence of NLOS errors leads to multipath effects during positioning, causing a substantial decrease in the accuracy of the Chan algorithm, significantly as indoor obstacles increase [17]. This highlights the ongoing challenge of developing robust, accurate indoor positioning systems in complex environments. The battery life of mobile devices and power wastage are also hot topics of concern [18].

In the current landscape, several companies and teams have developed analogous products with similar functionalities. In 2013, researchers at FTR Systems designed a robotic caddy named CaddyTrek, employing sensor technology to achieve the dynamic following of moving targets. However, widespread adoption has proven challenging. In 2017, the COWAHOBOT team developed an intelligent suitcase called COWAHOBOT R1, equipped with radio-frequency positioning technology, laser radar technology, and pressure sensor technology. It features functions such as human recognition and automatic following positioning. Due to its high cost, this smart suitcase faces difficulties in achieving broad accessibility. In 2019, the Italian company Piaggio introduced an autonomous cargo-carrying robot named Gita, utilizing camera sensor technology to construct three-dimensional maps for localization and tracking functionalities. However, these products primarily incur elevated costs and are designed for general users, lacking personalized adjustments for vulnerable, elderly, and disabled populations. They are not suitable for assisting these specific demographic groups in their daily lives.

The mobility of special populations, such as the elderly, disabled individuals, and pregnant women, is fraught with challenges, mainly when it involves carrying items or maneuvering luggage. These difficulties are further exacerbated in scenarios requiring navigation around obstacles like stairs, steps, plaza stairs, and entrance halls. Moreover, traditional autonomous following mobile IoT devices often falter in the face of such impediments and are hindered by diffuse reflection and NLOS conditions during localization and following processes. Addressing these multifaceted challenges, our study introduces an innovative mobile IoT device that combines following, obstacle negotiation, and material-handling functionalities. This device is grounded in UWB positioning technology. It harnesses the strengths of Gaussian, median, and mean filtering, integrated through a weighted fusion approach in conjunction with a BP neural network. We propose a novel UWB-based HFWF-BP neural network localization and following algorithm tailored to adapt to diverse scenarios and tackle the complexities of varied operational environments. Research and application of this technology, from a societal perspective, can significantly assist modern societies in addressing the challenges posed by population aging. Moreover, it can serve as a pivotal component in the development of barrier-free facilities for smart cities, aiding policymakers in formulating more-comprehensive social policies and contributing positively to overall societal advancement. From the standpoint of user groups, it has the potential to enhance the quality of life for the elderly, disabled individuals, pregnant women, and children in their daily lives. Simultaneously, it can mitigate the inconveniences associated with the lifting or carrying of heavy objects, promoting convenience and reducing the cost of living, while providing heightened safety assurance.

**Figure 1:**
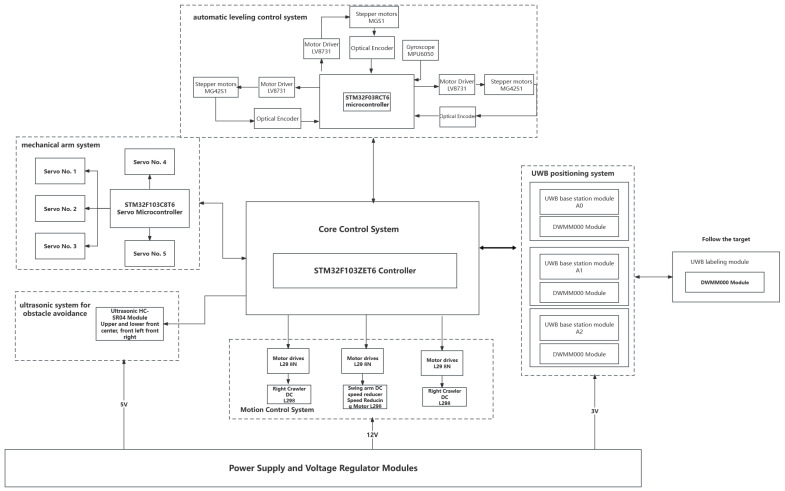
Structure of mobile IoT device following system.

We conducted secondary development on the DW1000 chip and designed a UWB positioning system. Initially, hardware calibration and experimental environment setup were performed. Subsequently, measurement values were obtained by calculating the TDOA, and hyperbolic equations were constructed. The three-sided positioning method was employed to determine the initial values of the target UWB tag. UWB measurement data were collected, and the obtained data were input into the HFWF-BP neural network localization algorithm to locate the moving target and obtain UWB measurement values. By integrating geometric positioning, the optimal tracking results were obtained. The performance evaluation of the experiment was conducted through dynamic/static CDF analysis. On mobile IoT devices, the HFWF-BP neural network algorithm was utilized to compute the distance and angular deviation from the UWB tag to the center of the UWB base station. The PID algorithm was employed for adjustment to control the PWM value, thereby regulating the motion of the autonomously tracking mobile IoT device. In this research, we designed an articulated autonomous positioning and following mobile IoT device based on UWB technology, which encompasses four major systems: an autonomous following system, an automatic leveling control system, a mechanical arm system, and an ultrasonic obstacle avoidance system. The mobile IoT device utilizes a UWB module to design the autonomous following system, following the moving target through a configuration of three UWB base stations and one UWB tag. The mechanical arm and ultrasonic obstacle-avoidance systems are responsible for detecting and overcoming or circumventing obstacles encountered during the following process. The automatic leveling control system corrects the tilt angle while overcoming obstacles, achieving the automatic leveling function. In the overall framework of the positioning and following system depicted in Figure 1, the DecaWave DWM1000 chip was used for the positioning module [19]; the STM32F103ZET6 chip serves as the main control chip of the system [20]; the motor drive module utilizes the L298N chip produced by the SGS Company [21]; a voltage-stabilizing module ensures stable current for each module.

**Figure 2:**
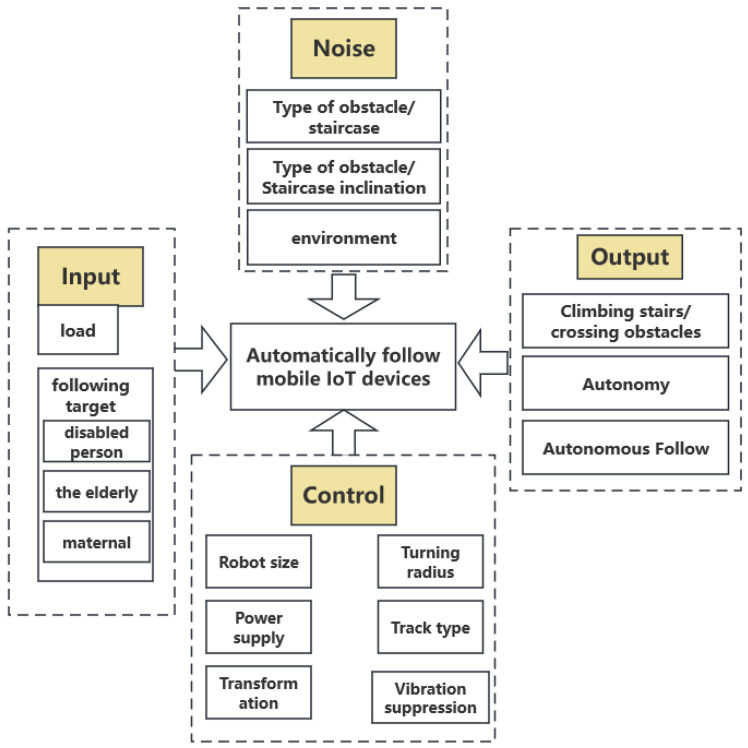
Parameter map of following mobile IoT device.

As illustrated in Figure 2, to delineate the design structure, we developed a Parameter Diagram for the autonomous positioning and following mobile IoT devices, which includes input, output, noise, and control components. The input consists of individuals who have difficulty moving, such as the elderly, disabled, and pregnant women, as well as items that meet the motor torque load requirements. The output is the capability to climb stairs, overcome obstacles, and control the carrying platform to maintain self-balance. Various noise factors and control elements must also be considered in practical tests.

## 2. Hybrid Filtering Localization Algorithm

### 2.1. Time Difference of Arrival Positioning Model

The Time Difference of Arrival (TDOA) model is solved to obtain the target location by measuring the arrival time difference between two base stations and the mobile target [22]. Let (x,y) denote the position of the mobile station (MS), $\left(x_{i},y_{i}\right)$ denote the position coordinates of the *i*-th base station $\left(BS_{i}\right)$, *c* be the propagation speed of the airwaves, and $R_{i1}$ denote the difference in the distance between the MS and $BS_{i}$ and $BS_{1}$, then:

(1) $$\begin{array}{c} R_{i1}=c\cdot \left(t_{i}-t_{1}\right)=R_{i}-R_{1}=\\ \sqrt{{\left(X_{i}-x\right)}^{2}+{\left(Y_{i}-y\right)}^{2}}-\sqrt{{\left(X_{1}-x\right)}^{2}+{\left(Y_{1}-y\right)}^{2}}\\ i=2,3,\cdots ,N \end{array}$$

Linearization can be obtained:

(2) $$R_{i1}^{2}+2R_{i1}R_{1}=K_{i}-2X_{i1}x-2Y_{i1}y-K_{1}$$

which is $\;X_{i1}=X_{i}-X_{1},Y_{i1}=Y_{i}-Y_{1}$; (i=2,3,⋯,N); N is the number of base stations.

### 2.2. Weighted Hybrid Filtering Localization Model

The sources of error in the positioning and following system of mobile IoT devices include antenna errors of the DWM1000, multipath errors, and NLOS errors, among others [23]. The fixed values within the chip largely determine the multipath errors, which originate from the transmission and reception mechanisms of UWB signals. In practical environments, obstructions can lead to signal energy loss. Therefore, by integrating filtering algorithms, the system can eliminate outlier data points, smoothing the overall system error and keeping it within an acceptable range.

**Figure 3:**
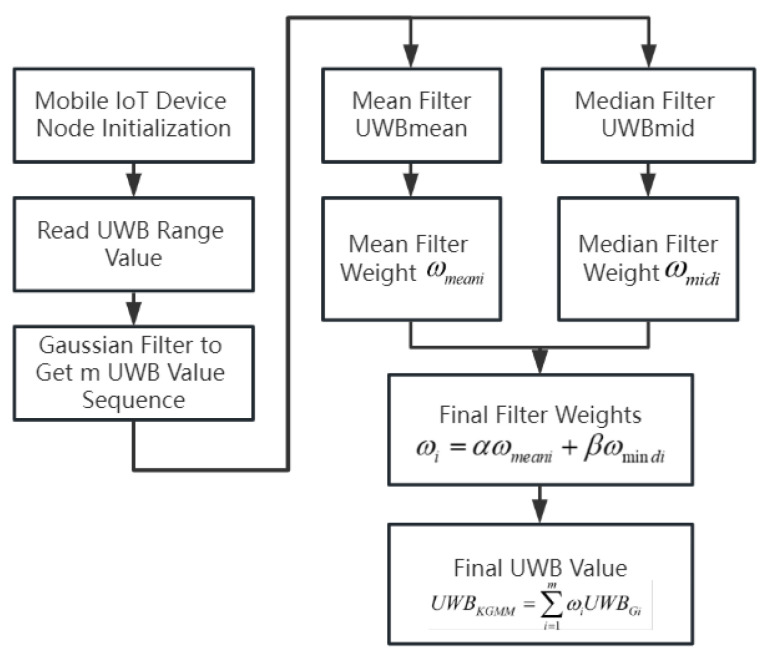
Technology roadmap for weighted hybrid filtering algorithm.

The singular application of filtering techniques does not yield optimal results in data processing [24]. Therefore, this research synthesizes the strengths and weaknesses of various filtering methods, combining Gaussian, mean, and median filters in a complementary manner to process UWB measurement data. This approach led to the development of a hybrid filtering weighting algorithm, the process of which is illustrated in Figure 3.

The proposed hybrid filtering weighting algorithm is as follows:

Step 1: The UWB information-receiving module reads a set of UWB measurements. These measurements are then processed using a Kalman filter to eliminate outliers.

Step 2: Gaussian filtering is applied to the dataset for secondary smoothing, resulting in refined m sets of UWB data groups.

Step 3: The filtered UWB data groups are sorted in ascending order, sequentially arranged as $U_{G1}$, $U_{G2}$, and ⋯, $U_{Gm}$. m represents the count of remaining UWB measurements from which the mean $U_{mean}$ and median $U_{mid}$ values are derived.

Step 4: For each UWB measurement, the mean-squared deviation from the mean and median values is calculated and used as a threshold. If the squared deviation is less than this threshold, the threshold value itself is used to determine the weight. Otherwise, the squared deviation is used to ascertain the optimal weight. The formula for calculating the weight is as follows:

(3) $$\omega_{meani}=\frac{\frac{1}{1+C_{1i}}}{\sum_{i=1}^{n}\frac{1}{1+C_{1i}}}$$

(4) $$\omega_{midi}=\frac{\frac{1}{1+C_{2i}}}{\sum_{i=1}^{n}\frac{1}{1+C_{2i}}}$$

which,

(5) $$C_{1i}=max\left\{\sum_{i=1}^{m}\frac{{\left(U_{Gi}-U_{mean}\right)}^{2}}{m},{\left(U_{Gi}-U_{mean}\right)}^{2}\right\}$$

(6) $$C_{2i}=max\left\{\sum_{i=1}^{m}\frac{{\left(U_{Gi}-U_{mid}\right)}^{2}}{m},{\left(U_{Gi}-U_{mid}\right)}^{2}\right\}$$

Step 5: Perform the following for $\omega_{meani}$ and $\omega_{mid}$: $\omega_{i}=a\times \omega_{meani}+b\times \omega_{midi}$, where a+b=1. Let each UWB measurement be multiplied with the weights $\omega_{i}$, after which, the sum is obtained to obtain the new weighted UWB measurements after correction.

(7) $$U_{GMM}=\sum_{i=1}^{m}\omega_{i}U_{Gi}$$

The hybrid filtering-weighted algorithm integrates the advantages of three distinct filtering methods, incorporating weights to eliminate outliers and smooth data by filtering edge values, ultimately yielding optimal positioning data. The proposed localization method in this article effectively suppresses interference in follow-up positioning caused by various factors, mitigating the jitter in UWB positioning data. The resulting UWB estimated values align more closely with the actual values, laying a solid foundation for subsequent integration with positioning and following algorithms and models.

### 2.3. Weighted Fusion Model

The ranging information of any node is subject to issues such as mean drift and large error fluctuations. From the perspective of the simulation model, this can be attributed to the UWB signal model exhibiting a clustered pattern. Within each cluster, the distribution conforms to a Poisson distribution, and the overall process aligns with a Poisson process. Additionally, human interference can lead to even more-significant ranging errors. Furthermore, UWB ranging information at short distances tends to be significantly less than the actual distance. This characteristic can lead to the decreased training effectiveness of the BP neural network, introducing biases in processing long-distance data. However, the ranging information processed through the Exponentially weighted moving average (EWMA) retains the characteristics of the original data and exhibits higher accuracy at longer distances. This can compensate for the deficiencies of the BP neural network. Therefore, weighted fusion is introduced, utilizing multiple measurement data sets to design the corresponding weighting functions. A cubic function form is employed to design the weighting coefficients, enhancing the overall effectiveness of the data-processing approach.

(8) $$U_{GMM}=\alpha U_{EWMA}+\left(1-\alpha \right)U_{GMM}$$

(9) $$\alpha =\frac{1}{2}\left[{\left(\frac{U_{EWMA}-\mu }{100}\right)}^{3}+1\right]$$

In Equation (8): ${\bar{U}}_{t}$ represents the range information after weighted fusion; $U_{EWMA}$ denotes the result of processing the original ranging data using the EWMA; $U_{GMM}$ is the data post-processing by the Gaussian median mean (GMM); α is the weighting coefficient, which is derived through empirical induction and fitting; μ is adjusted based on the sensor hardware specifications. To simplify the experiment, differences between base stations are not considered here and uniformly set to 520. The EWMA algorithm is as follows:

Exponentially weighted average formula:

(10) $$\nu_{t}=\beta \nu_{t-1}+\left(1-\beta \right)d_{t}$$

Exponentially weighted average formula with correction bias:

(11) $$U_{EWMA}=\frac{\nu_{t}}{1-\beta^{t}}$$

Equation (11) $\nu_{t}$ refers to the processed data from the EWMA during a cold start, which is adjusted using Equation (5) to correct the initial data deviation caused by the cold start, obtaining $U_{EWMA}$; β represents the rate of the weighted decrease, with a larger value indicating a slower rate of decrease, generally taken as 1 >β≥ 0.9. $U_{t}$ is the actual distance measurement information at time t.

## 3. BP Neural Network Localization Algorithm

### 3.1. NLOS Error Model

Let $r_{m}\left(t_{i}\right)$ be the measured value of the distance between the unknown node M and the m anchor node at time $t_{i}$. In a typical positioning environment, it is considered to be the sum of the system ranging error $n_{m}\left(t_{i}\right)$, the accurate distance $L_{m}\left(t_{i}\right)$, and the NLOS error $NLOS_{m}\left(t_{i}\right)$. Therefore, Equation (12) is as follows:

(12) $$r_{m}\left(t_{i}\right)=n_{m}\left(t_{i}\right)+L_{m}\left(t_{i}\right)+NLOS_{m}\left(t_{i}\right)$$

where $n_{m}\left(t_{i}\right)$ follows a normal distribution with parameter $N\left(\mu ,\sigma^{2}\right)$ and $NLOS_{m}\left(t_{i}\right)$ denotes the extended delay induced by the propagation of radio waves in the NLOS, obeying the COST259 model.

### 3.2. Chan Algorithm

Indoor mobile IoT devices typically require the acquisition of two-dimensional positioning data. In this study, three UWB base stations were positioned within the same horizontal plane in space. Utilizing the Chan algorithm, the coordinates of the target were calculated.

(13) $$d_{2,1}^{2}+2d_{2,1}d_{1}=K_{2}-2x_{2,1}x-2y_{2,1}y-K_{1}$$

(14) $$d_{3,1}^{2}+2d_{3,1}d_{1}=K_{3}-2x_{3,1}x-2y_{3,1}y-K_{1}$$

The associative Equations (13) and (14):

(15) $$\begin{array}{c} \{x=a_{1}+b_{1}d_{1}\\ \{y=\;a_{2}+b_{2}d_{2} \end{array}$$

where:

$$a_{1}=\frac{\left(K_{1}-K_{2}\right)y_{3,1}-\left(K_{1}-K_{3}\right)y_{2,1}+d_{2,1}^{2}y_{3,1}-d_{3,1}^{2}y_{2,1}}{2(x_{3,1}y_{2,1}-x_{2,1}y_{3,1})}$$

$$b_{1}=\frac{d_{2,1}^{2}y_{3,1}-d_{3,1}^{2}y_{2,1}}{x_{3,1}y_{2,1}-x_{2,1}y_{3,1}}$$

$$a_{2}=\frac{\left(K_{1}-K_{2}\right)x_{3,1}-\left(K_{1}-K_{3}\right)x_{2,1}+d_{2,1}^{2}x_{3,1}-d_{3,1}^{2}x_{2,1}}{2(y_{3,1}x_{2,1}-y_{2,1}x_{3,1})}$$

Substituting the above Equation (15) into the above Equation (1) yields a quadratic equation about $d_{1}$, and solving for the positive root is the estimated positional value.

### 3.3. BP Neural Network Error Correction

Choosing a different model or algorithm for a specific problem is a great challenge for researchers [25]. The back propagation (BP) neural network can solve nonlinear problems through learning data, encompassing two primary processes: forward propagation of signals and backward correction of errors [26]. The robust nonlinear approximation ability of the BP neural network is utilized to mitigate NLOS errors, thereby enhancing positioning performance. Typically, the hidden layer of the BP neural network employs a Sigmoid continuous function as the transfer function, enabling the implementation with a three-layer BP neural network. Given the often significant magnitude differences in the sample of input vectors, it is essential to normalize the input and output vectors. This normalization facilitates computation and prevents neurons from reaching an excessively saturated state, as illustrated in Equation (16):

(16) $$U^{*}=\frac{U_{GMM}-U_{GMM-min}}{U_{GMM-max}-U_{GMM-min}}$$

In Equation (16), $U^{*}$ is the normalized TDOA value, $U_{GMM-min}$ is the minimum value of the TDOA in the weighted fusion sample, and $U_{GMM-max}$ is the maximum value of the TDOA in the weighted fusion sample.

**Figure 4:**
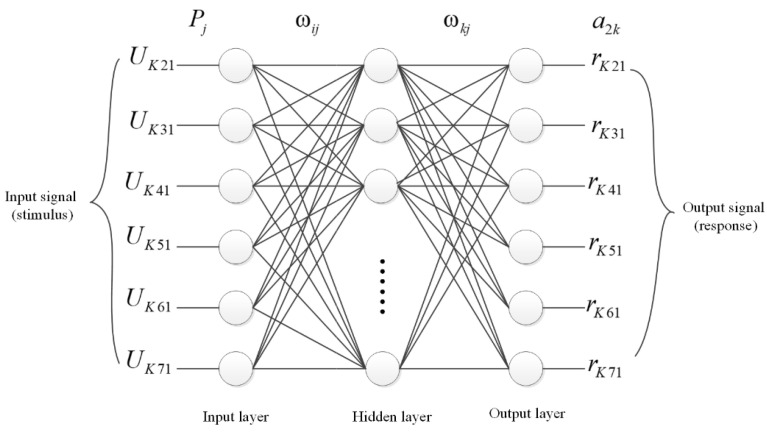
BP neural network model for TDOA error correction in NLOS environment.

By integrating the TDOA measurements from multiple base stations, this study corrects for NLOS errors, followed by position estimation using the Chan algorithm, thereby achieving higher localization accuracy in NLOS environments. Figure 4 presents the BP neural network model used for correcting TDOA measurements in NLOS environments provided by three base stations. The BP neural network comprises input, hidden, and output layers. The input layer comprises seven TDOA measurements provided by three correlated base stations.

The input vector is:

$$\bar{X}=\left[U_{k21,}U_{k31,}U_{k41,}U_{k51,}U_{k61,}U_{k71}\right]$$

The number of neurons in the hidden layer can be determined by an empirical formula, which is $N_{2}\geq$ lbT, where $N_{2}$ represents the number of neurons in the hidden layer, and T is the dimensionality of the training samples. Increasing the number of neurons in the hidden layer can enhance localization accuracy, but this comes at the cost of increased computational load. Considering both the efficiency and accuracy of the algorithm and after multiple rounds of experimentation, the number of nodes in the hidden layer of this network was established as seven. The transfer function for the hidden layer employs a Sigmoid function, $f_{1}\left(x\right)=tanh\left(x\right)$, which accepts any value as the input and produces an output value that ranges between −1 and +1.

The output layer comprises six neurons, employing a linear transfer function, Purelin, denoted as $f_{2}\left(x\right)=kx$, for its operation. The output of this layer is the adjusted TDOA values. The output vector is expressed as follows:

$$\bar{Y}=\left[r_{k21},r_{k31},r_{k41},r_{k51},r_{k61},r_{k71}\right]$$

### 3.4. Weighted Fusion BP Neural Network Localization Algorithm

**Figure 5:**
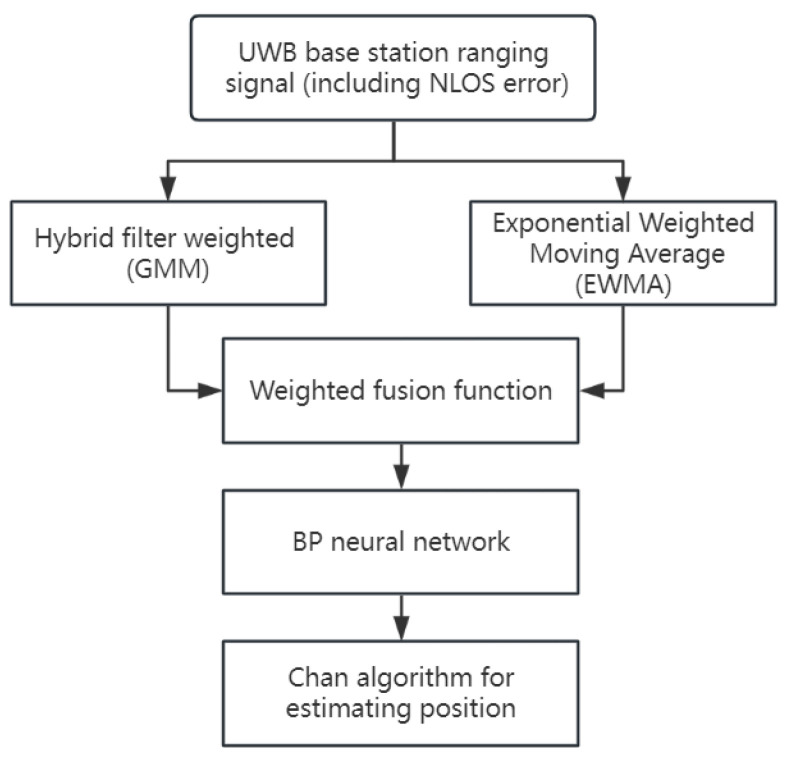
Localization Flowchart.

The Chan algorithm exhibits minimal error in TDOA measurements, particularly under the ideal conditions of zero-mean Gaussian random variables, where it achieves high localization accuracy. However, its performance is significantly affected in NLOS environments, where TDOA measurements are prone to more-significant errors [27]. By employing a WF-BP neural network to correct TDOA measurement data, the NLOS errors within TDOA measurements can be reduced, thereby effectively enhancing the localization accuracy when using the Chan algorithm for positioning. This study integrates the WF-BP neural network algorithm with weighted fusion, calculating the estimated values through the Chan algorithm, as illustrated in Figure 5.

The specific steps of localization using the WF-BP neural network are as follows:

Step 1: Assume K sets of TDOA values are measured in an NLOS environment. The measurement data set is processed using hybrid filtering for smoothing, and weights are introduced to obtain optimized values.

Step 2: A weighted fusion BP network is established to correct NLOS errors and is subsequently trained. TDOA measurements without NLOS errors are used as target sample vectors for network training. Normalize $\bar{X}$, and use it as the input for the BP neural network. Each set of TDOA values contains n TDOA data points. The normalized TDOA data of n reliable nodes and each anchor node under ideal conditions (i.e., without NLOS errors) are used as the output of the training samples. The aforementioned BP neural network is trained with these samples.

Step 3: The trained BP neural network is used to correct the TDOA measurement data, and the output obtained is the corrected normalized TDOA values.

Step 4: Post-correction normalized TDOA values are used in the Chan algorithm for position estimation.

## 4. Weighted Hybrid Filter Following Algorithm

**Figure 6:**
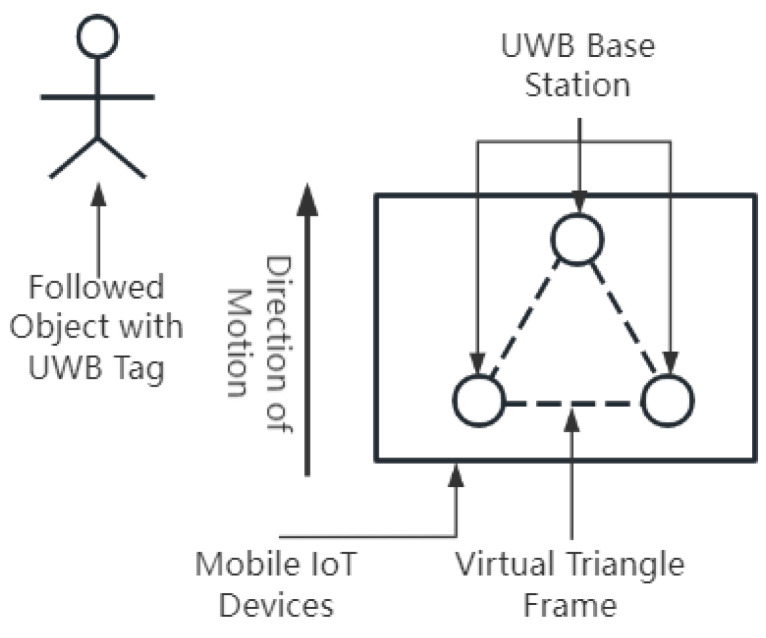
Schematic Diagram of autonomous following mobile IoT device.

Utilizing the weighted fusion BP neural network algorithm proposed in this article, a more-accurate following trajectory is achieved in conjunction with geometric positioning techniques. Three DWM1000 sensors were strategically placed vertically, forming an equilateral triangle on the mobile IoT device and serving as positioning bases. These selected sensors were fixed at the apexes of an equilateral triangle. The human subject and the mobile IoT device operated in the same environment. An appropriate DWM1000 sensor was chosen to be worn by the individual being followed, functioning as a mobile UWB tag, as illustrated in Figure 6.

**Figure 7:**
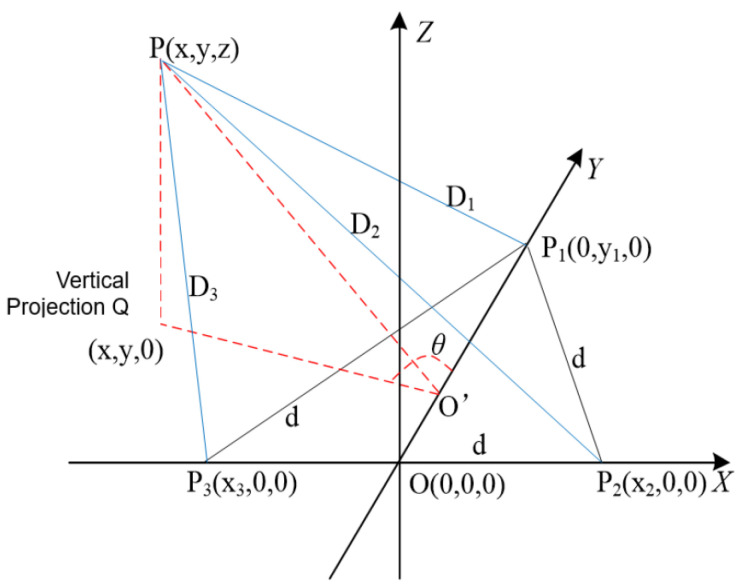
Schematic of the spatial location of the UWB base stations and the UWB tag.

In the three-dimensional space, the mobile IoT device functions as a coordinate platform. Utilizing a set of fixed base stations arranged in an equilateral triangle as the reference system, the XYZ spatial Cartesian coordinate system is established. The midpoint of the $P_{2}P_{3}$ side of the equilateral triangle is designated as the origin $O\left(0,0,0\right)$. The three vertices of the equilateral triangle serve as the coordinates, respectively. The coordinates of the tag worn by the object being tracked are denoted as $P\left(x,y,z\right)$. The direction of motion of the mobile IoT device is consistently aligned with the positive direction of the Y axis, as illustrated in Figure 7.

Assume that the lengths of the sides $P_{1}P_{2}$, $P_{2}P_{3}$, and $P_{1}P_{3}$ in Figure 7 are all d, the height of the equilateral triangle $P_{1}P_{2}P_{3}$ with the base side $P_{2}P_{3}$ is $OP_{1}$, and the projection height of $OP_{1}$ from the UWB tags to the plane of the fixed base station is PQ, $O^{\prime }$, and let $O^{\prime }$ be the center of the three fixed base stations of the equilateral triangle; the distance between the IoT device and the followed object is $PO^{\prime }$. Assuming that the target being followed is initially locked by the mobile IoT device at a specific moment, the setup involves placing three UWB positioning base stations at the vertices of an equilateral triangle. The distance is calculated by transmitting and receiving UWB signals between these base stations and the mobile UWB tag. After processing through the weighted fusion BP neural network algorithm, analytical geometric calculations yield the line connecting the tagged object P being followed and the center point $O^{\prime }$ of the equilateral triangle as follows:

(17) $$\begin{array}{c} L=PO^{\prime }=\sqrt{\frac{\left(D_{1}^{4}+D_{2}^{4}+D_{3}^{4}-D_{1}^{2}\times D_{2}^{2}-D_{2}^{2}\times D_{3}^{2}\right)}{3\times d^{2}}} \end{array}$$

The angle between the positive directions of the $PO^{\prime }$ and *Y* axes is

(18) $$\begin{array}{c} \theta =∠PO^{\prime }P_{1}=arctan\left(\frac{\sqrt{3}\left(D_{3}^{2}-D_{2}^{2}\right)}{D_{2}^{2}+D_{3}^{2}-2\times D_{1}^{2}}\right) \end{array}$$

Consequently, the position coordinates of the tracked object can be represented as $\left(L,\theta \right)$, thereby determining the relative position information of the tracked object to the mobile IoT device. This position information is updated in real-time, with the cumulative error being nearly negligible, ensuring the acquisition of the optimal following estimation.

**Figure 8:**
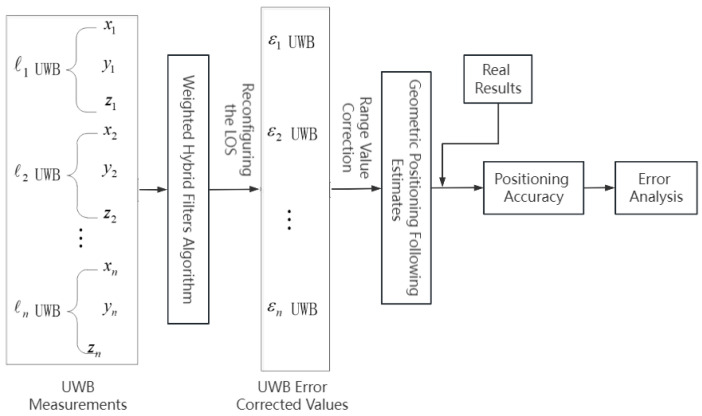
Weighted hybrid filtering follow algorithm roadmap.

In complex environments, factors such as NLOS and multipath errors inevitably affect the positioning and following performance of the mobile IoT device. The study integrates the advantages and disadvantages of various filtering algorithms, introduces weighting factors, and incorporates these into a weighted fusion approach. This is combined with the BP neural network algorithm to derive new UWB measurement values. The optimal following results are obtained by reconstructing the Line of Sight (LOS) UWB measurements and integrating geometric positioning. The performance of the positioning and following system was then analyzed to evaluate the effectiveness of the following mechanism, as illustrated in Figure 8.

## 5. Experiments and Analysis of Mobile IoT Device Localization and Following

### 5.1. Experimental Testing and Analysis of Mobile IoT Device Localization

#### 5.1.1. Hybrid Filtering Localization Analysis

**Figure 9:**
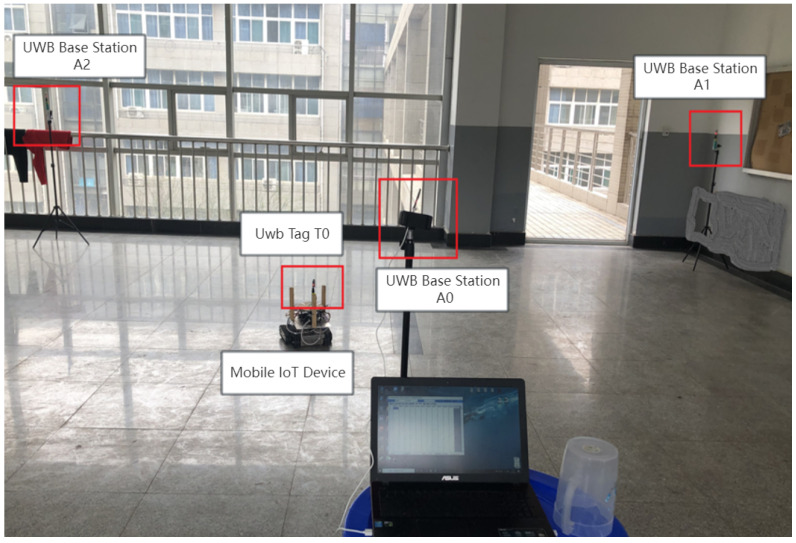
Live view of mobile IoT device localization experiments.

In the indoor environment, three fixed base stations were established with coordinates designated as A1 (0 m, 0 m), A2 (5 m, 6 m), and A3 (0 m, 10 m). A mobile object carrying a UWB tag moved constantly from the starting point at coordinates (1 m, 1 m) to the endpoint at coordinates (8 m, 5 m). The real-time distances measured by the three base stations to the positioning tag enabled the determination of the actual location of the mobile IoT device. Assuming that we neglect weather-related factors such as wind speed and humidity, the real-time positioning experiment of the mobile IoT device is illustrated in Figure 9.

**Figure 10:**
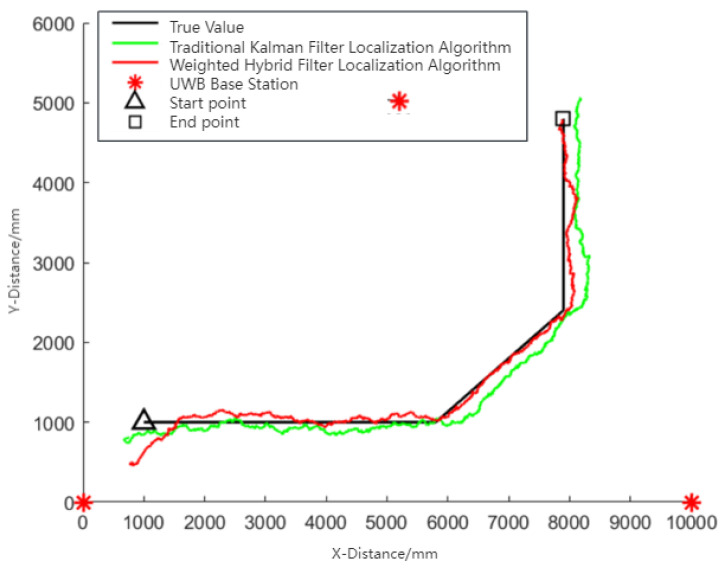
Mobile IoT device movement and localization trajectories.

**Figure 11:**
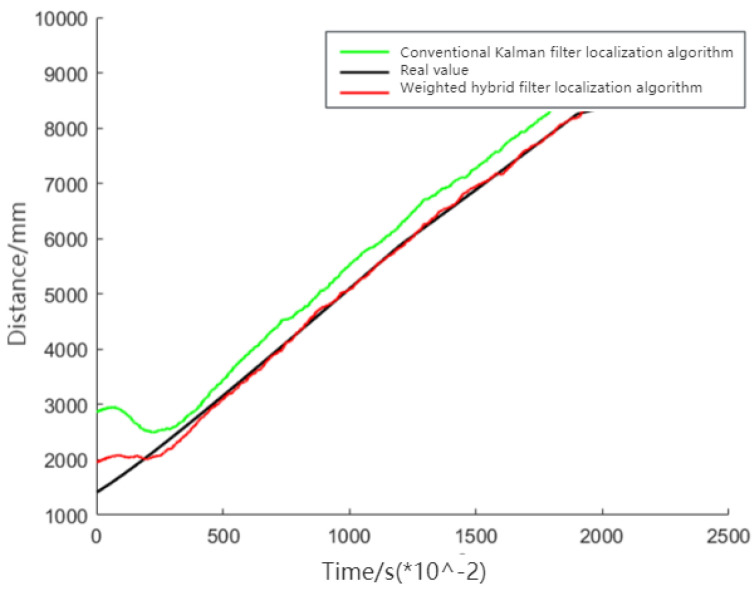
Measured distance between a mobile IoT device and the first UWB base station.

**Figure 12:**
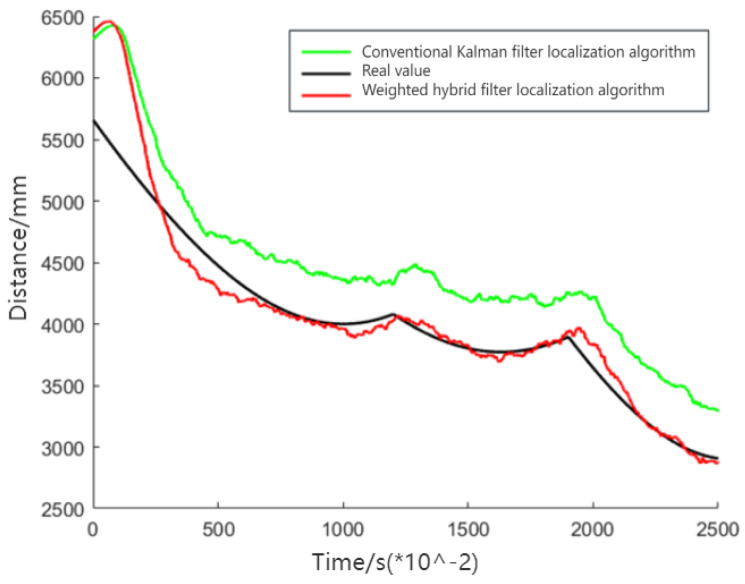
Measured distance between a mobile IoT device and the second UWB base station.

**Figure 13:**
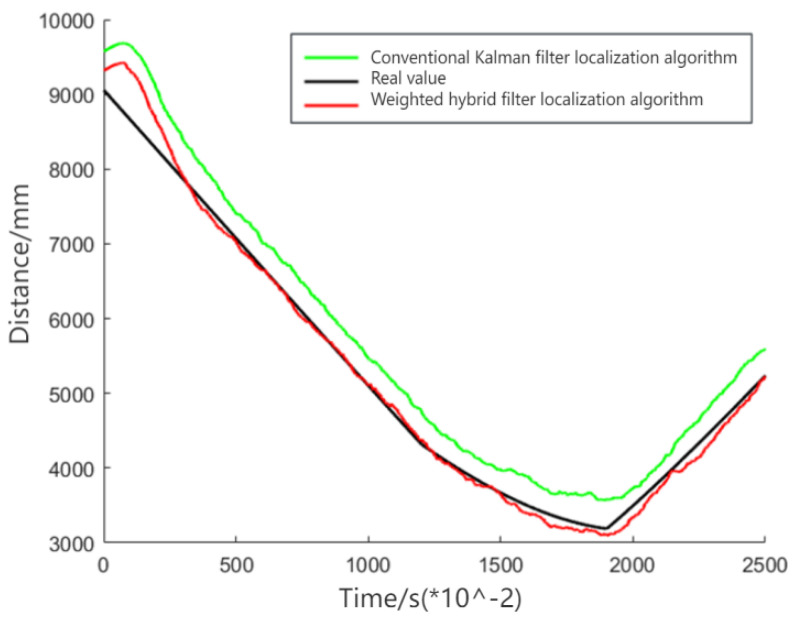
Measured distance between a mobile IoT device and the third UWB base station.

**Figure 14:**
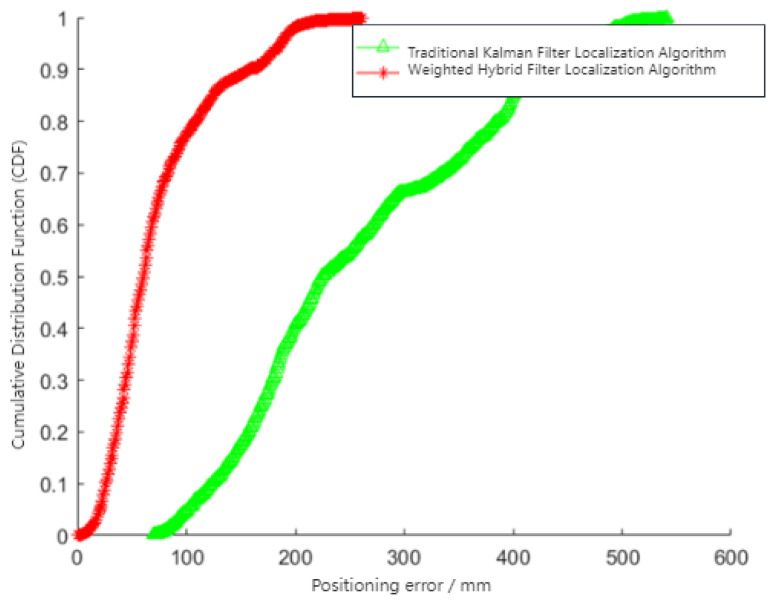
Mobile IoT device localization error.

Figure 10 demonstrates that the weighted hybrid filtering algorithm yields positioning estimates closer to the actual values than the traditional Kalman filtering positioning algorithm. This is particularly evident in the analysis of distances to various base stations, as shown in Figure 11, Figure 12 and Figure 13. The Cumulative Distribution Function (CDF) value indicates the frequency of positioning values within a certain error margin; a higher CDF value suggests better positioning performance [28]. As depicted in Figure 14, at a positional error of approximately 20 cm, the CDF value for the traditional Kalman filtering positioning algorithm is 45%, whereas for the weighted hybrid filtering positioning algorithm, it is 93%. This indicates that the weighted hybrid filtering approach achieves more-accurate positioning estimates.

#### 5.1.2. Experimental Analysis of BP Neural Network Localization

**Figure 15:**
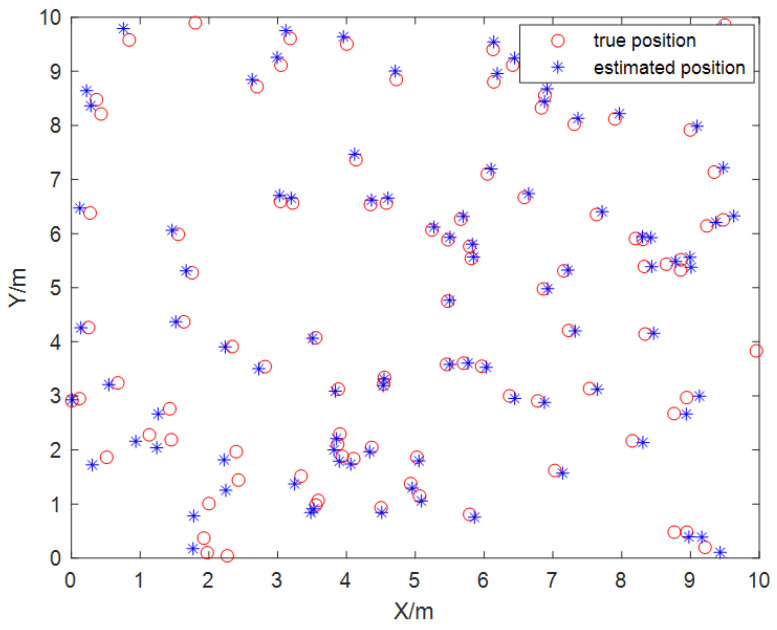
Localization results of weighted fusion BP neural network.

**Figure 16:**
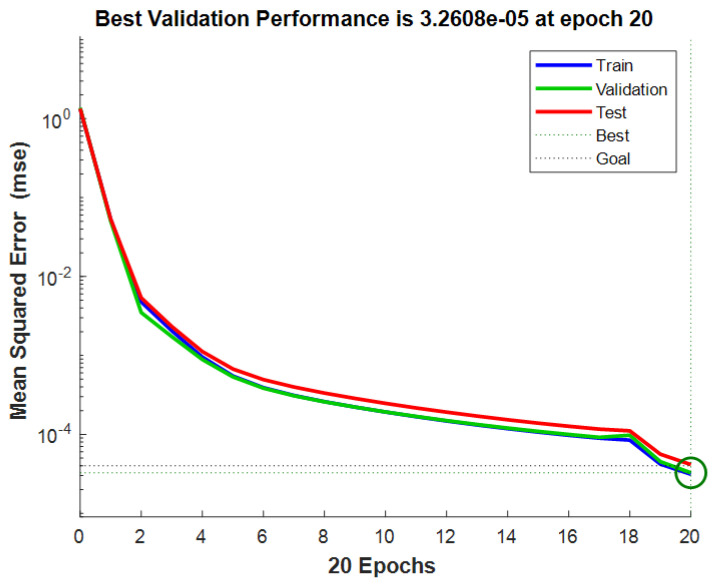
Network training error variation curve.

**Figure 17:**
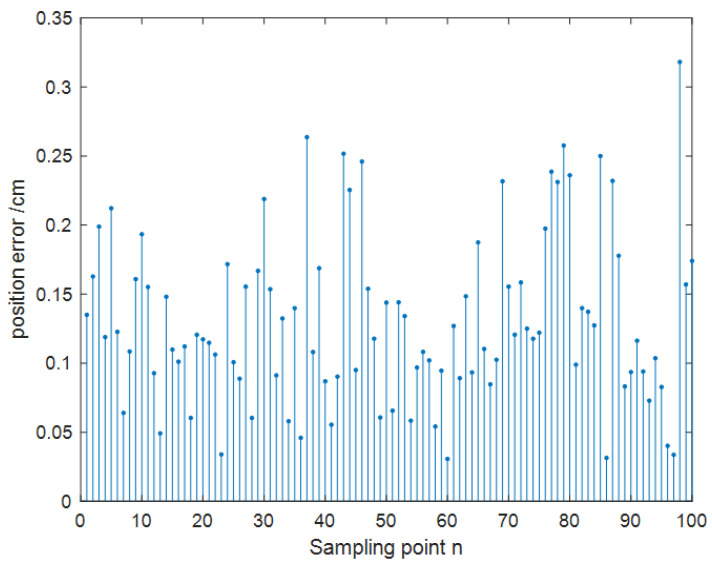
Localization error.

**Figure 18:**
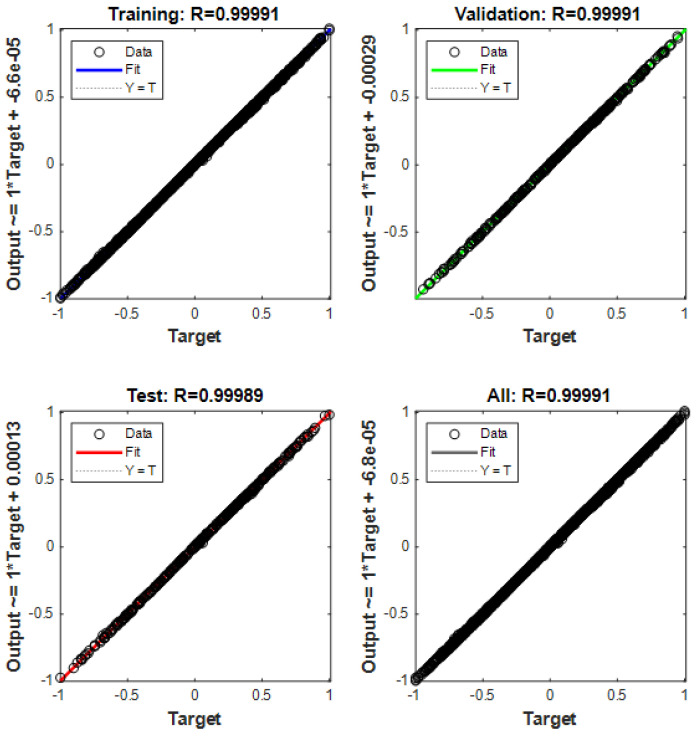
Regression analysis results.

The collected dataset of 1000 samples was divided into two parts: 900 samples for training and 100 samples for testing. Through network training, it was determined that the optimal number of nodes in the hidden layer was 7. The maximum number of iterations was set to 100, with a learning rate of 0.1. Predictions were made on 100 test data sets. Figure 15 shows that the training predictions for the localization results were conducted in a 10 × 10 m² two-dimensional space. The network convergence trend, depicted in Figure 16, indicates that convergence occurred at the 20th step. As illustrated in Figure 17, the prediction error of the localization results reveals that, upon this convergence, the input of any 100 sets of TDOA values resulted in a localization error of less than 0.15 cm in 95% of the cases. The regression analysis results, shown in Figure 18, demonstrate that the correlation coefficients (R) for the training set, validation set, test set, and the entire dataset were all greater than 0.99, indicating a good fit for the model.

#### 5.1.3. Weighted Fusion BP Neural Network Localization Analysis

**Figure 19:**
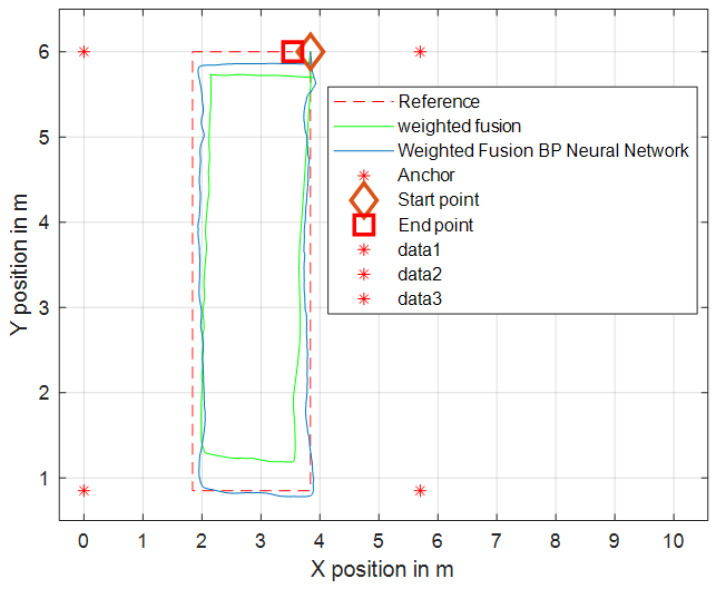
Weighted fusion of BP neural network trajectory records.

**Figure 20:**
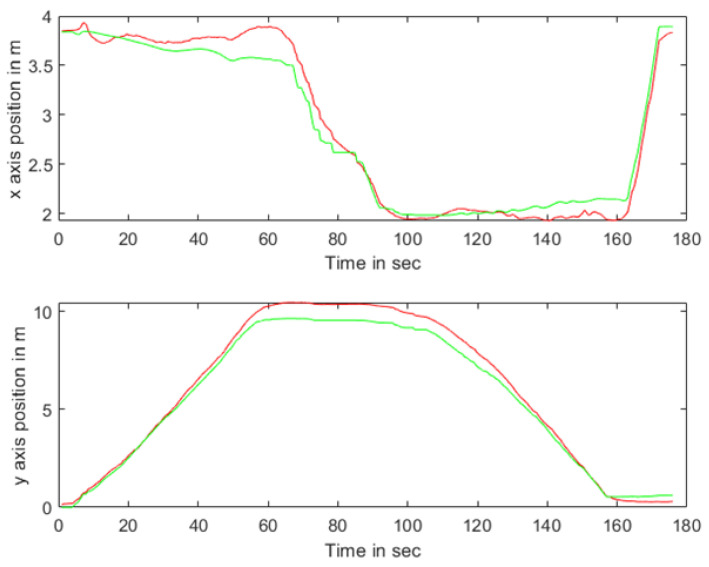
Error in X and Y directions of weighted fusion BP neural network.

Under the same environmental conditions depicted in Figure 9, four base stations were arranged within a 7 × 10 m^2^ two-dimensional space, where NLOS errors were introduced. The positioning results, as corrected by the WF-BP neural network algorithm, demonstrated a significant improvement in rectifying the NLOS errors. This is evident from the enhanced alignment with the actual positions, as illustrated in Figure 19 and Figure 20.

### 5.2. Experimental Testing and Analysis of Mobile IoT Device Following

**Figure 21:**
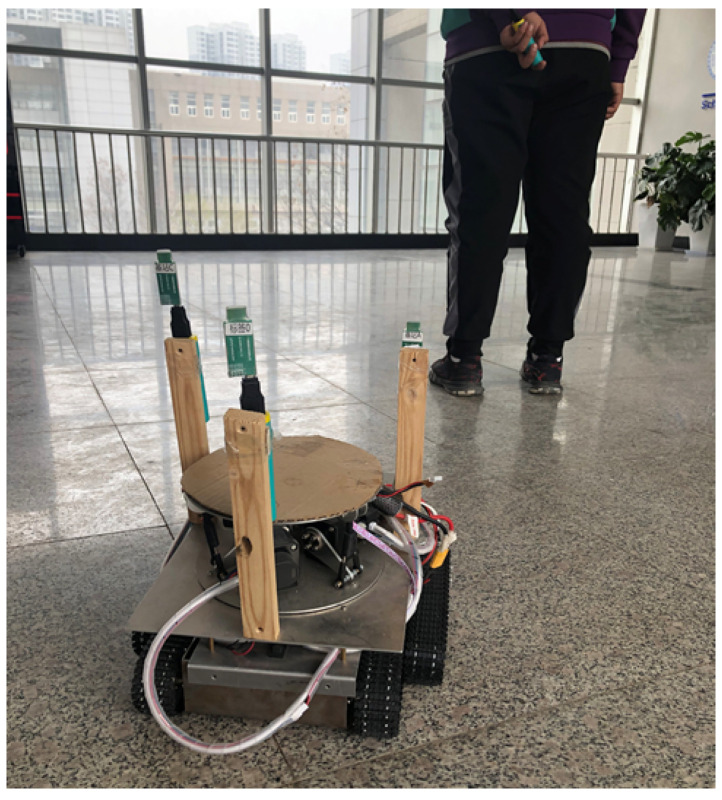
Mobile IoT device following experiment photo.

**Figure 22:**
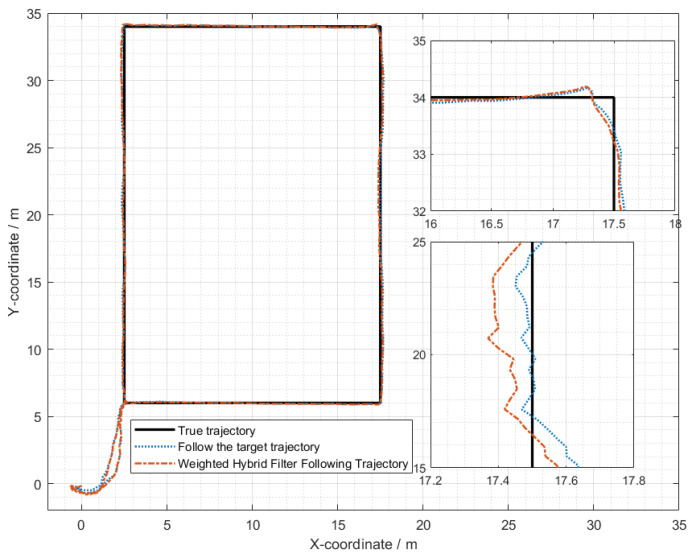
Comparison with weighted hybrid filtering algorithm.

**Figure 23:**
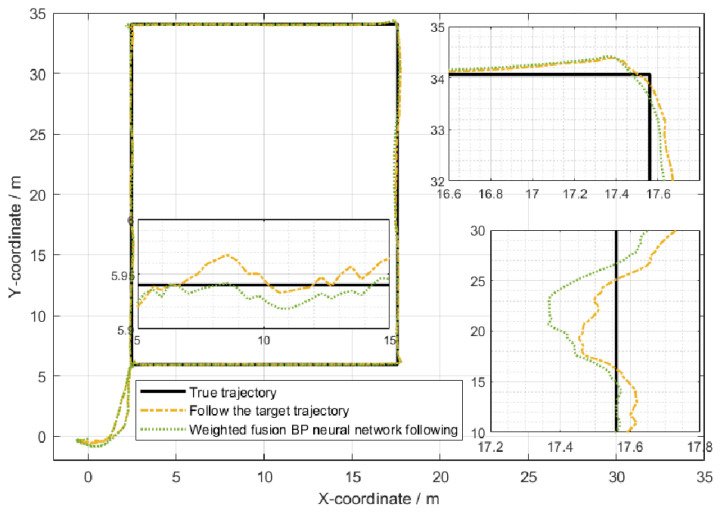
Comparison with weighted fusion BP neural network following algorithm.

In the indoor setting, the following UWB tag and UWB base stations formed an equilateral triangle, with the straight-line distance between the center of this triangle and the UWB tag set at 1 m. The three base stations were positioned at a mutual distance of 0.3 m from each other. Both the individual carrying the mobile UWB tag and the mobile following IoT device were equipped with separate UWB positioning tags. Several UWB base stations were uniformly distributed within the indoor environment. The individual with the mobile tag moved at a walking speed (approximately 5 km/h), and his/her position and that of the additional tag were determined through the base stations distributed across the experimental site. The movement trajectories of the individual carrying the mobile tag and the mobile following IoT device are illustrated in Figure 21. The WF-BP neural network proposed in this paper was employed to analyze the moving targets, with the following trajectories depicted in Figure 22 and Figure 23.

**Figure 24:**
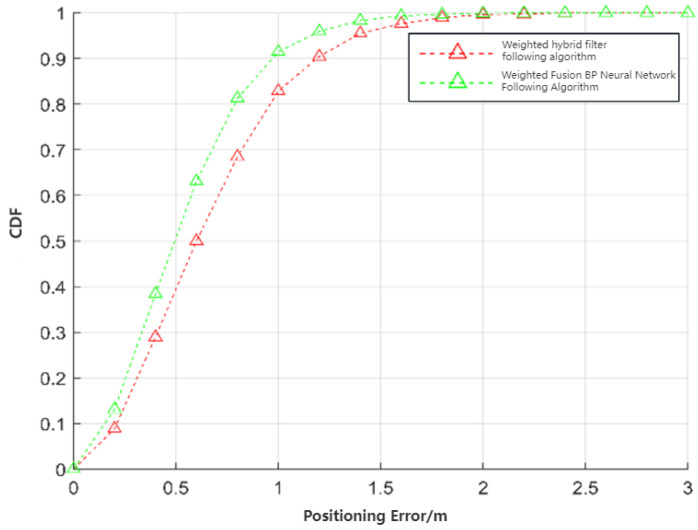
Estimation errors of two following algorithms.

Figure 22 and Figure 23 illustrate that both following algorithms can achieve accurate autonomous tracking recognition and are less prone to losing track with mobile IoT devices. However, the WF-BP neural network algorithm demonstrated a higher trajectory alignment with the target than the traditional Kalman filter algorithm, resulting in more-effective following performance. It was observed that the mobile IoT devices exhibited less precision in following accuracy at turns, leading to significant trajectory deviations in some curved paths, yet without any instances of target loss or substantial deviation from the following path. Through comprehensive comparison and analysis, we observed that the HFWF-BP neural network localization algorithm exhibited higher algorithmic complexity compared to the algorithms it was benchmarked against. This resulted in slight delays in controlling the mobile IoT device. Simultaneously, the mobile IoT device, as shown in Figure 21, adopted a tracked drive and steering mechanism for stability, contributing to increased steering errors and reduced sensitivity. Consequently, these factors introduced some degree of error in the experiments. These issues could be mitigated by upgrading hardware or employing alternative auxiliary steering mechanisms, such as omnidirectional wheels (e.g., mecanum wheels), to further enhance performance. As depicted in Figure 24, the WF-BP neural network algorithm showed superior following precision compared to the traditional Kalman filter algorithm, better fulfilling practical requirements and every day following services. In a static environment, the distance between the follower and the three base stations was set to 1 m, with a testing duration of 100 s. Both following algorithms were employed for static ranging during this period to determine the UWB measurement error, as shown in Figure 17.

**Figure 25:**
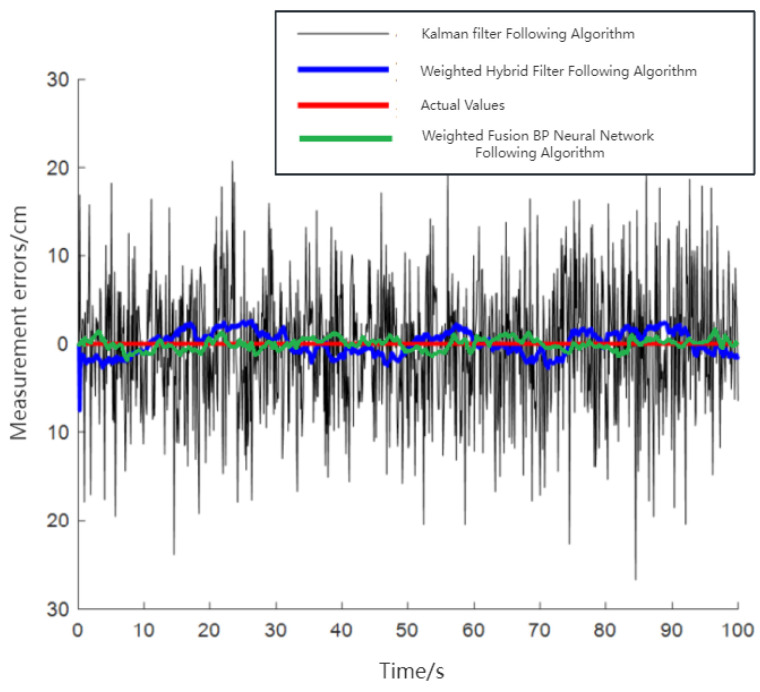
Static ranging UWB measurement error.

As observed in Figure 25, the traditional Kalman filtering following algorithm demonstrated that most range estimation errors were concentrated around 8 cm. This included disturbances such as UWB jitter values and systematic errors. In contrast, the WF-BP neural network following algorithm showed that most of the measurement estimation errors were centered at 4 cm. Moreover, these errors were relatively more stable, better fulfilling the practical requirements for accurate positioning and tracking.

The comparison presented in Table 1 reveals that the HFWF-BP neural network localization algorithm, proposed in this study, exhibited notable improvements compared to various technologies/algorithms despite not having the lowest device cost and deployment difficulty. Remarkably, it represents a significant enhancement over computer vision. Simultaneously, this algorithm achieved ultra-high centimeter-level positioning accuracy. The experimental outcomes, as depicted in the relevant figures, affirmed the effective and stable performance of both tracking algorithms with mobile IoT devices. Notably, the WF-BP neural network algorithm showcased superior trajectory alignment compared to the traditional Kalman filter algorithm, resulting in more-efficient tracking capabilities. Despite minor deviations during turns, the mobile IoT devices exhibited consistent accuracy in following, avoiding target loss or significant path deviations. Upon in-depth analysis, it became evident that the HFWF-BP neural network localization algorithm introduced higher algorithmic complexity, resulting in slight delays in device control. The use of a tracked drive and steering mechanism, as illustrated in the figures, contributed to increased steering errors and reduced sensitivity. Addressing these challenges through hardware upgrades or alternative steering mechanisms, such as omnidirectional wheels like mecanum wheels, could enhance the overall system performance. Further insights underscored the superior precision of the WF-BP neural network algorithm in following compared to the traditional Kalman filter algorithm, making it more suitable for practical applications and everyday tracking services. In a static environment with specified conditions, both tracking algorithms underwent static ranging, revealing that the WF-BP neural network following algorithm demonstrated stable and accurate measurement estimation errors, fulfilling practical requirements for precise positioning and tracking.

## 6. Conclusions

This paper establishes an experimental platform based on UWB technology and designs a model for autonomous tracking of mobile IoT devices. It introduces an improved TDOA positioning algorithm based on UWB, termed the HFWF-BP neural network positioning algorithm. This approach integrates the characteristics of Gaussian filtering, median filtering, and mean filtering methods. Leveraging the neural network’s capability to approximate arbitrary nonlinear mappings and its fast learning properties, the algorithm effectively overcomes the deficiencies present in traditional TDOA positioning Chan algorithms, making it more suitable for complex multipath environments. The simulation results indicated that, through neural network correction of NLOS errors, the algorithm exhibited stronger suppression capabilities against NLOS errors. Compared to traditional Chan algorithms and Kalman filtering algorithms, the proposed algorithm demonstrated higher positioning accuracy and reliability. Its performance surpassed that of the Chan algorithm, and due to its simplicity and effectiveness, the UWB-based TDOA positioning improvement algorithm utilizing neural networks holds significant practical value. Potential applications include, but are not limited to, advanced autonomous navigation in smart cities, precise tracking in industrial settings, and enhanced safety measures in healthcare environments. This research not only addresses current challenges in autonomous positioning, but also paves a promising path by utilizing UWB technology and innovative algorithms to enhance the precision and adaptability of IoT devices in real-world scenarios.

## 7. Potential Application Scenarios and Future Works

Automatic tracking IoT devices with low power consumption, strong anti-interference capabilities, and high positioning accuracy have extensive application prospects across various domains. They can significantly contribute to improving the overall quality of life for vulnerable groups. These devices can assist in daily tasks, such as fetching items, providing companionship, or offering support in various activities. By reducing dependence on external assistance, individuals can experience increased autonomy and a higher quality of life. Furthermore, the deployment of mobile IoT devices can serve as a crucial component in emergency response systems. These devices can be equipped to carry essential life-saving equipment, such as Automated External Defibrillators (AEDs) and necessary medications. In critical situations, these mobile devices can swiftly navigate through a designated area, providing immediate access to crucial medical resources. This application not only increases the chances of saving lives during emergencies, but also exemplifies the versatile and impactful role that mobile IoT devices can play in enhancing community well-being.

Beyond addressing the needs of an aging population and individuals with disabilities, this research can be applied to an automatic tracking shopping cart system in supermarkets, enabling obstacle avoidance and seamless following of consumers throughout their shopping journey. In the field of waste management, autonomous tracking cleaning vehicles can navigate independently, allowing workers to focus on their tasks, reducing workload, enhancing efficiency, and effectively preventing dust generation during construction processes. Intelligent epidemic prevention and disinfection robots can autonomously follow healthcare professionals or patients with infectious diseases, automatically disinfecting areas they traverse, effectively curbing the spread of infectious diseases and safeguarding public health. In outdoor environments, this technology holds significant potential. For instance, in the realm of intelligent transportation, the HFWF-BP neural network UWB positioning technology can be utilized for precise positioning and real-time tracking of vehicles, pedestrians, and other modes of transportation. This enhances traffic efficiency, reduces accidents, and contributes to the development of smart transportation and cities.

In future research, given the limitations of the BP neural network algorithm, such as slow convergence, susceptibility to local optima, challenges in determining hidden layer node quantities, and oscillations during training, we plan to incorporate the momentum effect and adaptive learning rates for improvement, optimizing the performance of the BP neural network. The introduction of momentum and adaptive learning rates will refine the BP neural network, adjusting weights to enhance training robustness. Additionally, we will explore advanced sensor technologies, such as millimeter-wave radar and stereoscopic vision systems, to comprehensively and accurately capture environmental information. By integrating data from multiple sources, we aim to enhance the perception capabilities of the automatic tracking device in dynamic environments, thus strengthening its applicability in complex settings, continuing to advance hardware technology is a priority, aiming to reduce device size, weight, and power consumption while enhancing portability and lifespan. This effort will contribute to the broader and more-effective deployment of automatic tracking IoT devices in practical applications, promoting their widespread use in areas such as smart cities and intelligent transportation. Finally, we aim to apply the research findings more extensively in social services, particularly addressing the personalized needs of vulnerable groups, elderly individuals, and those with disabilities. Through customized design and intelligent adjustments, automatic tracking devices can better meet the diverse requirements of different user groups, enhancing their sustainability and universality in the realm of social services.

## Figures and Tables

**Table 1:** Comparison of technologies/algorithms in various Aspects.

	Technology and Algorithm	Accuracy	Range	Device Price	Deployment Difficulty
References [7,8]	Computer vision	Highly affected by the environment	Depends, typically within 5 m	Expensive	Difficult and restricted
Reference [9]	UWB	30–40 cm	50–100 m	Moderate cost	Average
Reference [10]	Kalman filter UWB	20–30 cm	50–100 m	Moderate cost	General
Reference [12]	Wireless LAN (WiFi)	50–60 cm	10–20 m	Low cost	Easy to deploy
Reference [13]	Weighted K-nearest neighbors and Kalman filtering	Around 20 cm	50–100 m	Moderate cost	Average
This research	HFWF-BP neural network localization algorithm	10 cm	50–100 m	Moderate cost	Average

## Data Availability

Data are contained within the article.

## Abbreviations

The following abbreviations are used in this manuscript:

IoT	Internet of Things
NLOS	Non-Line of Sight
LOS	Line of Sight
UWB	Ultra-Wideband
HFWF-BP	hybrid filtering weighted fusion back propagation
WF-BP	weighted fusion back propagation
RMSE	Root-Mean-Squared Error
TDOA	Time Difference of Arrival
EWMA	Exponentially weighted moving average
GMM	Gaussian median mean
BP	back propagation
CDF	Cumulative Distribution Function
